# Experiences of Discrimination Among Women and Gender Diverse Veterans Using Veterans Health Administration Health Care

**DOI:** 10.1089/heq.2024.0085

**Published:** 2024-09-30

**Authors:** Jodie G. Katon, Samantha K. Benson, Vyshnika Sriskantharajah, Lisa S. Callegari, Karissa Fenwick, Kristen E. Gray, P. Adam Kelly, Ashley C. Mog

**Affiliations:** ^1^ Center for the Study of Health Care Innovation, Implementation, and Policy, VA Greater Los Angeles Health Care System, Los Angeles, California, USA.; ^2^ Center for Innovation in Veteran-Centered and Value-Driven Care, VA Puget Sound Health Care System, Seattle, Washington, USA.; ^3^ Department of Obstetrics and Gynecology, University of Washington School of Medicine, Seattle, Washington, USA.; ^4^ Department of Health Systems and Population Health, University of Washington School of Public Health, Seattle, Washington, USA.; ^5^ Southeast Louisiana Veterans Health Care System, New Orleans, Louisiana, USA.; ^6^ Department of Medicine, Tulane University School of Medicine, New Orleans, Louisiana, USA.

**Keywords:** Veterans, women, discrimination, bias, marginalization

## Abstract

**Introduction:**

Women Veterans are diverse in terms of racial, ethnic, and gender identities and sexual orientation and may experience a variety of forms of discrimination and stigma in health care settings. Our objective was to understand discrimination experienced by women Veterans in the context of Veterans Health Administration (VA) care.

**Methods:**

We analyzed data from a series of semistructured telephone interviews with Veterans identified as females in the VA medical record who received VA health care in the past 12 months, purposively sampled by race/ethnicity and age (*N* = 28). The interview guide elicited experiences with VA health care, including discrimination. Interviews were audio-recorded, transcribed, and analyzed using inductive and deductive content analysis.

**Results:**

We identified themes regarding structural discrimination, interpersonal discrimination, and strategies employed in response to discrimination. Veterans described structural discrimination, including challenges with spaces not designed to accommodate disabilities or safety needs and care not sensitive to their gender, trauma histories, or sexual orientation. Interpersonal discrimination included harassment from other Veterans and biased treatment from VA providers and staff based on gender, appearance, and sexual orientation. Gender-based discrimination compounded across additional axes of marginalization including body size and stigma regarding mental illness. Experiences of discrimination undermined Veterans’ sense of belonging and trust in VA and created barriers to accessing care. Veterans engaged in various strategies to protect themselves from discrimination and get needed care.

**Discussion:**

Quality improvement efforts that address the experience of women Veterans using VA health care must consider multiple forms and sources of discrimination and the intersection of gender-based discrimination with other forms of marginalization.

## Introduction 

Discrimination, or the unfair treatment of socially defined subordinate groups, functions to reinforce relations of dominance and subordination and the privileges of the dominant group or groups.^1,2^ Discrimination in health care operates on multiple levels, including structural and interpersonal levels.^3^ Structural discrimination refers to discrimination and bias built into processes, policies, and physical spaces, such as the inclusion of race adjustments in clinical algorithms or clinical spaces not accessible for wheelchair users.^1,4,5^ Interpersonal discrimination, in contrast, involves biased or unfair treatment based on the implicit or explicit assumptions or beliefs of one individual about one or more identities (or presumed identities) of another individual.^1^ Discrimination when seeking care through the Veterans Health Administration (Veterans Affairs [VA]) may be of particular concern to women Veterans who are the fastest growing group of new VA users but remain a numeric minority in VA (∼8% of VA users).^6^

Early research identified significant gender disparities in VA care and outcomes, including preventive care and screenings and chronic disease risk reduction and management.^7^ Additionally, studies revealed a high prevalence of women Veterans experiencing gender-based harassment from other Veterans in addition to gender-based discrimination from VA staff and providers.^8–11^ Notably, women Veterans frequently exist at the intersection of multiple forms of stigmatization and marginalization, which can have profound implications for their experiences of VA health care and health outcomes. Veterans identified as female in the VA medical record are increasingly diverse in terms of their racial and ethnic identities, gender identity, and sexual orientation. As of fiscal year 2018, 29% of women Veterans identified as Black or African American, and 7.2% identified as Hispanic or Latina.^6^ VA is estimated to be the largest provider of health care for Lesbian, Gay, Bisexual, Transgender, Queer, plus (LGBTQ+) individuals globally, although exact numbers are difficult to estimate due to historic limitations in the electronic health record and stigma due to the prior Don’t Ask, Don’t Tell policy.^12^ Women Veterans using VA health care also have a high prevalence of larger body size and mental illness, which are frequently stigmatized in health care settings.^13–15^ Thus, efforts to address gender-based harassment and discrimination in VA health care must consider the ways in which women Veterans experience marginalization in multiple, intersecting ways.^16–20^ For example, Black women Veterans may experience dismissal or judgment from health care providers due to misogynoir, or the unique form of misogyny experienced at the intersection of racism and sexism.^16,21–25^

Outside VA, discrimination in health care based on gender, race, body size, disability, and LGBTQ+ identity is well documented, with ∼20% of health care users in the United States experiencing interpersonal discrimination in the context of health care.^26^ Although the majority of existing literature on discrimination experienced by women Veterans in VA health care focuses exclusively on gender-based discrimination, studies also document experiences of gendered racism and discrimination based on sexual orientation or gender minority status.^8–10,23,27–29^ Most of this literature is limited to specific types of VA health care (e.g., contraceptive care, gynecological care), and exclusively focuses on interpersonal discrimination. Thus, there is a gap in the literature with respect to understanding structural discrimination women Veterans experience in VA and how women Veterans may experience structural and interpersonal discrimination in unique ways due to their positionality at the intersection of multiple axes of marginalization. Our objective in this analysis was to understand different forms of discrimination experienced by women Veterans in the context of VA care, including structural and interpersonal discrimination, using an approach that acknowledges how these experiences are shaped by intersecting axes of marginalization.^16,19,20,24,25^

## Methods

### Positionality statement

The research team consists of VA researchers and clinical practitioners committed to investigating and improving the care experiences of women/gender diverse Veterans, two of whom identify as having a disability. Most of our research team identify as women. All authors are users of non-VA health care in the United States. Among the three researchers directly involved in conducting interviews and coding these data, all identify as queer, cisgender women, and one identifies as non-White. None of the authors of this work are Veterans.

### Study design and population

This was a secondary analysis of a series of semistructured interviews completed as part of a larger VA-funded project to develop and validate a patient-reported experience measure of VA health care for women Veterans (HSR-IIR-21-104).^30^ We generated the original national sample from a cohort of Veterans identified as female in the VA administrative record who had used VA outpatient, inpatient, or community care (e.g., VA paid care from community providers) in the past 12 months; had a mailing address and phone number; did not have a diagnosis of dementia according to International Classification of Disease Version 10 (ICD-10) codes; and were not deceased. Four sampling strata were created stratifying by age (<45 years old, ≥45 years old) and race/ethnicity (non-Hispanic White vs. all other racial and ethnic identities).^30^ The sampling strata were chosen based on the demographics of women Veterans to try to ensure representation across age and racial/ethnic groups with different military and VA experiences. Approximately 40% of women Veterans who use VA are <45 years old and a similar percentage identify as belonging to a non-White racial or ethnic group.^13,31^ However, among those <45 a higher percentage identify as non-White race/ethnicity compared with those ≥45 years old. Therefore, the stratification was used to try to ensure the representation of non-White racialized women Veterans in the younger and older age groups.^31^ We also considered age an important determinant of Veterans’ experiences in VA due to varying health care needs by age, which might result in differing experiences of VA care (e.g., younger Veterans would be more likely to have sought contraception versus older Veterans who were more likely to need care for chronic conditions or need certain health screens like mammography or colonoscopy).

An equal number of Veterans within each stratum were randomly selected for recruitment and sent study information by mail or encrypted email. Veterans were given an opportunity to opt out of further contact from study staff either by a prepaid postcard for those randomized to mail recruitment or via a Qualtrics survey link embedded in the encrypted email. Those who did not opt out were contacted by telephone up to three times to gauge their interest in the study. To ensure representation of LGBTQ+ Veterans, we monitored participation rates of LGBTQ+ participants during the study and used inclusive recruitment materials. Given that at least one participant identified their gender as nonbinary, we use the term women/gender diverse Veterans to describe study participants going forward. Recruitment took place between June 2022 and January 2023 and was conducted on a rolling basis within each recruitment stratum until the analytic study team deemed that we had reached thematic saturation and adequate representation across strata.^30^ Participants received $50 upon completion of the interview to compensate them for their time. This study was reviewed and determined to be exempt by both the VA Puget Sound and VA Greater Los Angeles Institutional Review Boards.

### Data collection

The interview guide was designed to elicit rich narratives regarding Veterans’ experiences with VA care, including asking about domains of trust, safety, respect, privacy, communication, and discrimination (Supplementary Data S1), which were selected *a priori* based on a literature review of women/gender diverse Veterans’ health care experiences and the broader literature on patient-reported experience.^27,32–34^ Specific probes, grounded in verbatim participant language and informed by the *a priori* domains, were used throughout interviews.^35^ Trained qualitative interviewers conducted semistructured telephone interviews, which were audio-recorded and transcribed, removing identifying details. At the end of the interview, participants completed a brief demographic questionnaire to provide key sample descriptors.

### Analysis

We conducted analysis and collected data concurrently. Two coders used ATLAS.ti version 8 software and a combination of deductive codes based on interview guide questions and *a priori* domains alongside inductive coding of emergent concepts to categorize the data.^36,37^ We ensured transcripts matched the audio recording before coding. After co-coding a subset of transcripts, coders independently coded and took analytic notes in memos, noting observations, questions, and assumptions for discussion. Coders met weekly with the core analytic team to discuss interviewer reflections, review analysis memos, refine the codebook, verify the generated themes, and assess when thematic saturation (reaching depth of understanding and data repeating across interviews) was achieved to ensure the trustworthiness and credibility of findings.^38,39^ We maintained detailed records of analytic and reflexive discussions to establish an audit trail for dependability and confirmability.^38,40^ The methodological process and analytic team for this secondary analysis did not differ from the primary analysis and study aim. Given the depth of the data under the *a priori* domain of discrimination, we employed targeted coding to fully explore the experiences of discrimination reported by our Veteran sample beyond the scope of the original study aim.^41^ Exemplar quotes were chosen that best illustrated identified themes and are provided below along with the participants age, self-identified race, location of the experience, and study ID (e.g., 47, Black, VA, [D18]).

## Results

A total of 28 interviews with a mean duration of 42 min (median: 42, range = 19–60) were completed. The majority of respondents (75%) described experiencing discrimination in the context of VA care (Table 1). Most of these experiences were described in response to our opening interview question regarding a memorable experience of VA care. While nearly all participants also described positive experiences with VA, their experiences of discrimination continued to influence how they viewed VA and the way in which they approached VA care. We identified themes regarding structural discrimination, interpersonal discrimination, and strategies employed in response to discrimination.

**Table 1.** Interview Length and Self-Reported Characteristics of All Interviewees and Those Endorsing Discrimination of Any Kind

	Total interviewed, *N* = 28	Total endorsed discrimination, *N* = 21
Interview length (min), median (range)	42 (19–60)	48 (19–60)
Veteran characteristics		
Hispanic/Latina ethnicity, *n (%)*	6 (21%)	5 (24%)
Race, *n* (%)		
Black	8 (29%)	5 (24%)
White	18 (64%)	15 (71%)
Other	1 (4%)	1 (5%)
More than one race	1 (4%)	0 (0%)
Age, *n* (%)		
≤34	6 (21%)	3 (14%)
35–49	13 (46%)	11 (52%)
50–64	7 (25%)	5 (24%)
≥65	2 (7%)	2 (10%)
Gender identity, *n* (%)		
Woman	27 (96%)	20 (95%)
Nonbinary	1 (4%)	1 (5%)
Sexual orientation, *n* (%)		
Straight or heterosexual	21 (75%)	14 (67%)
Lesbian or homosexual	2 (7%)	2 (10%)
Bisexual	2 (7%)	2 (10%)
Asexual	2 (7%)	2 (10%)
Prefer to self-describe	1 (4%)	1 (5%)
Ever deployed, *n* (%)	13 (46%)	8 (38%)
VA care ever received, *n* (%)		
Primary care	27 (96%)	20 (95%)
Mental health care	23 (82%)	17 (81%)
Reproductive health care	19 (68%)	13 (62%)
Specialty care	25 (89%)	19 (90%)

VA, Veterans Health Administration

### Structural discrimination

Veterans described having to get care in physical environments that did not meet their physical or safety needs and this was sometimes exacerbated by lack of gender-sensitive care or lack of awareness of the needs of women/gender diverse Veterans.

Physical spaces: Veterans recounted efforts to access VA care in spaces not designed to accommodate Veterans’ disabilities. This included check-in processes that were not accessible for a blind Veteran and clinic entrances that did not accommodate wheelchairs easily.

“Like they’re waving their hand, and I’m not seeing that… finally I hear somebody go, next please. And I start walking over, and I’m like where are you? And they go, we need you to slide your card. And I’m like, ok, where is the card slider thing?” 63, White, VA (C19)

“When I had to go get examined, once I would get into the room that was good. But the doorways, my friend had trouble getting the wheelchair through that little narrow doorway.” 47, Black, VA (D18)

Other Veterans described physical layouts in clinical spaces that did not take safety concerns of women/gender diverse Veterans into account:

“I’m an MST [military sexual trauma] patient, and they [VA] have only 1 phone [in the inpatient psychiatric ward], and I had to walk from the place that I was back in the female area, all the way across the hall, and the mess hall, and then yet another hall, and another hall, and stay there… but the patients there, they are worse, they are a little aggressive. So why they subjected a female to pass through that?” 52, Hispanic/Latina, VA (C3)

Lack of gender-sensitive care: Veterans also described experiences when care was not sensitive to their gender, trauma histories, or sexual orientation. For example, a Veteran who was a rape survivor described feeling re-traumatized when assigned to a PTSD therapy group attended primarily by male Veterans, while another described repeatedly having to “out” herself as standard processes assumed heterosexuality:

“I’m sitting in this room with these men [for group therapy]… And, you know, I didn’t say what I was most afraid of, but it triggered the whole thing in my mind, you know? Imagining all of those scenarios… And within 10 minutes I just, I left.” 63, White, VA (C19)

“Doctors assume that you’re straight, so you find yourself always having to out your sexual preference in order to get the information out clear. It’s always, are you practicing safe sex? Are you using contraception? Well no I’m not, because I’m in a same-sex relationship… Or, are you at risk of being pregnant? No I’m not. Well, either way we still have to do a urine on you because you could be pregnant. And I’m like, no I’m not, I’m a full blown lesbian, there’s no possible way I can be pregnant.” 33, White, VA and non-VA (A13)

### Interpersonal discrimination

Veterans described interpersonal discrimination from other Veterans, VA providers, and VA staff. Some of these experiences involved the intersection of gender and other axes of marginalization.

Harassment by other Veterans: Veterans experienced gender-based harassment from other Veterans, often promoted through enforced proximity in waiting rooms and other spaces.

“So when you’re stuck in the waiting room with them, they [men veterans] say inappropriate things. The last time I was there, one of the older men… he said, I just really don’t like women, at all.” 39, White, VA (A21)

Discrimination by providers and staff: Discrimination by VA providers and staff based on appearance and stereotypes led to the dismissal of Veterans’ health concerns and occasionally to overtly discriminatory comments by providers. Notably, these stereotypes frequently had gendered or racialized aspects to them although Veterans did not always explicitly tie their experiences to gender and/or race.

“…every time I go into the doctor’s office to complain about pain, or fatigue, or anything like that, they always say, well, you’re overweight. They don’t want to test anything, or do anything, because they’re like, you’re overweight.” 43, Black, VA (B12)

“This woman basically, she based her determination on my looks. I looked too decent to have PTSD. So I guess if I had went in with no makeup, hair not done, then I would have qualified. If she had been in the military and had some type of military background, she would have known as a woman with PTSD or anxiety, or suffering from depression, when we come out we try to mask it the best way we can.” 52, Black, VA (D27)

“I just felt she was a little rude. She was like, it sounded like she was making snide comments when we took my weight and my blood pressure. She was like, oh, a little heavy there. Or something like that she had said.” 29, Black, VA (B13)

Veterans also experienced providers who expressed homophobic opinions.

“I did let him [VA provider] know that I am a lesbian… I think more than once he mentioned, he said that I needed to go to church to find a good man. So I just felt uncomfortable after that. I think maybe I had one other visit with him and I think he made another comment similar to that, and I just did not want to go back to him after that.” 41, Hispanic/Latina, VA (B22)

Finally, Veterans described gender-based discrimination where providers dismissed their concerns and their expertise in their own bodies.

“I was like, is it possible for me to get into a women’s endocrinologist type of specialist? And she literally said no. Because, you’re a woman, your hormones change all of the time, they’re not going to do anything for you…I feel like that’s a little disrespectful for someone to be told, no, no, no, you’re a woman, your hormones are constantly going to change, they’re going to find nothing for you. I feel like it’s a little disrespectful because it makes you feel like, I know, I’ve been a hormonal person since I hit puberty, but something is not right…it’s diminishing a woman’s own intuition of her own body” 36, Hispanic/Latina, VA (B21)

### Responses to discrimination

Veterans described various strategies they employed in response to the discrimination they experienced in VA. Some tried to “be quiet and go through the paces when I’m there” (64, White, VA, [C18]) to get needed care. Others described forgoing needed care, avoiding certain areas or clinics, or using non-VA care when possible (e.g., leaving a VA therapy group within 10 min [C19]). Finally, Veterans described how they advocated for themselves, by doing research in advance or even paying for tests that VA providers were unwilling to order, reporting complaints to those who they trusted to take action, and organizing with other Veterans:

“I had to go outside, pay for a chiropractor to do my x-rays, because the VA didn’t do it. they kept telling me that I was overweight, that was the only problem… And I took those x-rays to the VA. And then that’s when they were like… we do see something wrong, now we’re going to do the x-rays here. And then I got services.” 43, Black, VA and non-VA (B12)

“I had a [VA] doctor that I was being seen by, a psychologist, and I did not like the care that I was getting from him. Especially comments he was making about my sexuality… I let my primary care doctor know about the comments that he made, and she annotated that in the records, and she actually followed up on that.” 41, Hispanic/Latina, VA (B22) 41

“Over the years it [gender-based harassment by male Veterans] has gotten a lot better. I used to work with a woman’s veteran organization that brought it to the higher ups at the VA” 43, Black, VA (B12)

## Discussion

A variety of forms of discrimination encountered while seeking VA care were reported within and across women/gender diverse Veterans. This included structural factors, such as lack of services sensitive to their needs and clinical spaces not designed to accommodate their physical disabilities or safety concerns as women/gender diverse Veterans. Experiences of interpersonal discrimination ranged from gender-based harassment from other Veterans to discrimination by staff and clinicians. In response to discrimination, Veterans engaged in various strategies to protect themselves and get the care they needed.

In terms of structural discrimination, Veterans described lack of access to or availability of clinical services specific to their needs, particularly as trauma survivors, such as women-only group therapy. Women/gender diverse Veterans also described having to navigate physical spaces such as waiting rooms or parts of inpatient facilities that were unsafe or hostile. Notably, women Veterans who perceive VA as not being sensitive to the needs of women Veterans are more likely to discontinue use of VA care and are unable to benefit from many of VA’s unique programs and services, leading to deepening gender disparities among Veterans.^42^ Women/gender diverse Veterans also experienced systems and processes that were unable to accommodate physical disabilities, such as blindness or wheelchair use. Structural ableism, or the cumulative and reinforcing ways in which willful or unconscious discrimination manifests in policy and institutions that prioritize or assume able-bodiedness, is linked to lower rates of preventive care and poorer health outcomes among those with disabilities.^5,43–48^ Findings are mixed regarding disability-related disparities in preventive health care and chronic disease management among women Veterans due to differing definitions of disability and potential unaccounted for variation between those using and not using VA.^49,50^ Research is needed that focuses on disability among women/gender diverse Veterans and accessibility of VA care, using methods and measures consistent with the most current recommendations and instruments.^51–53^

Women/gender diverse Veterans also described a variety of experiences of interpersonal discrimination from other Veterans, staff, and providers, including gender-based harassment and bias from providers based on identity or physical appearance. Experiences of gender-based harassment were consistent with previous reports in VA indicating that as many as 25% of women Veterans seeking VA care experience harassment from other Veterans and that LGBTQ+ women Veterans experienced higher rates of harassment.^8^ Recent evidence suggests that gender-based harassment at VA, while still a concern, may be decreasing, possibly due to efforts to enhance awareness and provide training to VA staff on intervening to prevent or stop harassment.^54^ Similarly, previous research has characterized negative interactions with VA providers regarding disclosure of LGBTQ+ identity.^55^ Although VA is making strides toward ensuring affirming care for all Veterans regardless of gender identity or sexual orientation there is still much work to be done.^56,57^ Notably, no Veterans described experiences of discrimination that they attributed to their age, this is in contrast to findings from a qualitative study of women Veterans <45 years old from minoritized racial and ethnic groups, which identified a theme of age creating challenges in terms clinician relatability and sometimes led to dismissal of health concerns.^58^

Finally, Veterans experienced interpersonal discrimination from providers based on their gender, appearance, and negative stereotypes regarding body size and mental illness. While participants did not necessarily recognize how these experiences involved multiple overlapping axes of marginalization, it is important to note that the discrimination they described based on body size or mental health stigma has gendered and racialized components. Specifically, fatphobia, which emerged in the 18th and 19th centuries, is rooted in othering and dehumanizing of Black female bodies.^59^ Similarly, stigma regarding mental illness is frequently gendered through the stereotype of the “mad woman.”^60,61^ Such discrimination is particularly salient to women/gender diverse Veterans’ experiences of VA care given the patriarchal and hierarchical nature of military culture, which frequently carries over into VA; the high proportion of women/gender diverse Veterans who identify as Black or African American; and the large prevalence of mental health conditions in this population.^6,62^ In response to these experiences, women/gender diverse Veterans engaged in various strategies to protect themselves and get needed care. Such strategies highlight their tenacity and resourcefulness but were not without time, financial, or emotional costs.

Strengths of this study include efforts taken to ensure diverse representation of women Veterans’ experiences in VA; recognition of both structural and interpersonal discrimination; and an analytic approach that explicitly acknowledged that gender-based discrimination is inextricably intertwined with other forms of discrimination (e.g., gendered racism).^16–20^ There are also some important limitations regarding our findings. Some of the experiences of discrimination that Veterans described may have occurred in the past and may not reflect recent changes that have occurred in individual facilities. However, regardless of how long ago these experiences were and other more positive or affirming experiences of VA care, they continued to shape Veterans’ perceptions of VA and VA care.^30^ Additionally, this was a secondary analysis of a series of interviews focused broadly on Veterans’ experiences with VA care. Thus, we may not have fully captured all salient themes related to discrimination that women/gender diverse Veterans experience in VA. Similarly, our sample size and sampling scheme did not allow for representation across all potential overlapping identities.

## Health equity implications

Our findings highlight various forms of structural and interpersonal discrimination women/gender diverse Veterans face in VA, which undermine Veterans’ sense of belonging and safety and trust in VA, potentially leading to delayed or missed care. Quality improvement efforts must consider multiple forms and sources of discrimination and address the intersection of gender-based discrimination with other forms of marginalization. Practices that reinforce patient bodily autonomy and ensure that all Veterans feel heard in clinical encounters can also help to improve Veteran experience and mitigate the impact of experiences of discrimination.^30^ As VA continues to update existing facilities and build new ones it is important to incorporate concepts of inclusive design that account for the safety and access needs of women/gender diverse Veterans. Continuing to invest in a racially diverse VA provider workforce and training all providers in race-conscious and gender-inclusive care is essential to ensure all Veterans receive safe and affirming care. Finally, new patient-reported experience measures are needed to monitor the effectiveness of quality improvement efforts among women/gender diverse Veterans as current measures used by VA (e.g., SHEP) do not fully capture discrimination that these Veterans experience in VA.

## Abbreviations Used

HSR: Health Systems Research
ICD: International Classification of Diseases
LGBTQ+: Lesbian, Gay, Bisexual, Transgender, Queer, plus
VA: Veterans Health Administration
