# Supplementary material for: Experiences of Discrimination Among Women and Gender Diverse Veterans Using Veterans Health Administration Health Care
Source: Health Equity. 2024 Sep 30;8(1):692–700. doi: 10.1089/heq.2024.0085 (PMC11512093; doi:10.1089/heq.2024.0085)
Supplement: Supplementary Data S1 [file heq.2024.0085_supp_datas1.pdf]

Interview Introduction/Preamble

During this interview, I'm interested in hearing about your experiences getting care at VA. Please feel free to talk about any kinds of healthcare you may have received from VA, including things like primary care, mental health care, specialty care, or reproductive healthcare. I'm interested in your experiences getting ***care in the community*** *that's covered by VA in addition to any care you might get a VA facility or clinic.*

We particularly want to hear about any positive, negative, or other memorable experiences you may have had being a patient, and what contributes most to your overall experience of the VA healthcare you receive.

Grounded Prompts

Grounded interviewing prompts: If responses are limited or require clarification, probes may be used to elicit more detailed responses. Probes should use words or phrases presented by the participant using one of the following formats:

- *What do you mean by _________?*
- *What else?*
- *Tell me more about ____________.*
- *When did (they, you, it, etc) _________?*
- *Give me an example of ____________.*
- *Where did (they, you, it, etc) ________?*
- *What happened next?*
- *Who____?*
- *Tell me about a time when ________.*
- *Was that a VA staff member, scheduler, your provider, a fellow Veteran, or someone else?*
- *Walk me through ______________.*
- *Which service was that?*
- *Tell me about another time__________.*

Interview Questions

1. Tell me about the care you receive at VA.

2. [If needed] Can you tell me about an experience getting care at VA that you remember well?

a. Tell me about getting the appointment scheduled.
b. Walk me through going to your appointment.
c. What, if anything, went well?
d. What, if anything, didn't go well?
e. What, if anything, made the experience easier?
f. Was there anything that made the experience difficult?
g. Tell me about the setup of the waiting room and exam room for your appointment.
h. [if needed] Tell me about another recent or memorable visit at VA

3. [If needed] Tell me about the people you interacted with when trying to get care for [service/concern].
   a. What worked well?
   b. What did not work well?
   c. Tell me about talking to your VA provider about [services/concerns].
   d. What, if anything, would you have wanted your provider to do differently?
   e. Are there any other times when you talked with your provider about [service/concern] and wished they had done anything differently?

TRUST

4. When receiving care at VA, have you trusted your providers?
   a. Give me an example of that.

5. Do you feel that your providers have trusted you?
   b. [if needed] For example, did your provider believe you about your symptoms or trust you to follow treatment instructions?
   c. Give me an example of that.

SAFETY

6. When receiving care at VA, what has made you feel safe (whatever that means to you)?

7. When receiving care at VA, has there been anything that made you feel unsafe (whatever that means to you)?
   a. Give me an example of that.
   b. [If applicable] Is there anything the VA could do to address this?

PRIVACY

8. Privacy can be important for people when they are getting healthcare. Tell me about your experience with privacy when you have gotten care at VA?

RESPECT

9. Can you describe a time when you felt respected getting care at VA?

10. Can you describe a time when you felt disrespected getting care for at VA?
   a. [If applicable] Is there anything the VA could do to address this?

DISCRIMINATION

11. Have you ever been treated differently at VA due to one or more of your identities or characteristics? For example, your race, sexual orientation, gender identity, weight or size.
   a. Give me an example of that.
   b. How, if at all, did that affect your care?
   c. [If applicable] Is there anything the VA could do to address this?

10. Have you ever been treated differently at VA due to one or more of your medical or mental health conditions [If needed: for example, your weight, PTSD diagnosis]?

a. Give me an example of that.
b. How, if at all, did that affect your care?
c. [If applicable] Is there anything the VA could do to address this?

GENERAL/WRAPPING UP

11. What would you want other Veterans to know about [service/care mentioned] at VA?

    a. Is there anything that the VA could do to improve care for Veterans like you?

12. Is there anything else we should know about your experience care at VA?
